# Development of a new ERA-CRISPR/Cas12a method for rapid sensitive detection of *Ralstonia pseudosolanacearum* in eucalyptus

**DOI:** 10.3389/fpls.2025.1706542

**Published:** 2025-11-25

**Authors:** Xiaoping Peng, Shengkun Wang, Yipin Zhang, Shengjie Wang, Sen Meng, Lipan Hu, Haibin Ma

**Affiliations:** 1 College of Biology and Food Engineering, Chongqing Three Gorges University, Chongqing, China; 2 State Key Laboratory of Tree Genetics and Breeding, Research Institute of Tropical Forestry, Chinese Academy of Forestry, Guangzhou, China

**Keywords:** *Ralstonia pseudosolanacearum*, ERA-CRISPR/Cas12a, eucalyptus, rapid detection, visualization

## Abstract

**Introduction:**

*Ralstonia pseudosolanacearum* is a significant pathogenic bacterium that causes bacterial wilt in Eucalyptus worldwide. Asymptomatic Eucalyptus cuttings may harbor substantial quantities of *R. pseudosolanacearum*, leading to latent infections that increase the risk of pathogen dissemination. Currently, there are no effective methods available to cure Eucalyptus bacterial wilt; therefore, rapid and sensitive detection methods for this disease are urgently needed to mitigate losses in the Eucalyptus industry.

**Methods:**

In this study, we developed a rapid and accurate diagnostic method for detecting *R. pseudosolanacearum* based on enzymatic recombinase amplification (ERA) combined with clustered regularly interspaced short palindromic repeats (CRISPR)/Cas12a technology.

**Results:**

The ERA-CRISPR/Cas12a method demonstrated high specificity and exhibited no cross-reactivity with other common bacterial pathogens. The detection limit for *R. pseudosolanacearum* by the fluorescence and the LFS detection system was as low as 10^0^ copies/µL. Furthermore, the results can be visualized through an ERA-CRISPR/Cas12a fluorescent signal (ERA-CRISPR/Cas12a-FL), color under blue light or an ERA-CRISPR/Cas12a lateral flow strip (ERA-CRISPR/Cas12a-LFS).

**Discussion:**

The newly developed ERA-CRISPR/Cas12a method could detect *R. pseudosolanacearum* in Eucalyptus rapidly and accurately. Moreover, the samples can be detected within one hour by our developed method, highlighting the significant potential for onsite applications in disease management.

## Introduction

The bacterial wilt in Eucalyptus was first reported in China in the early 1980s (Cao, 1982). Isolates obtained from diseased Eucalyptus in Asia were subsequently identified as *Ralstonia pseudosolanacearum*, belonging to phylotype I (Carstensen et al., 2017; Oliveira et al., 2023). Taxonomically, *Ralstonia solanacearum*, the causal agent of bacterial wilt, is a Gram-negative, soil-borne bacterial within the *Ralstonia solanacearum* species complex (RSSC), which comprises several closely related species, including *R. pseudosolanacearum*. Eucalyptus bacterial wilt poses major challenges for early detection because of its cryptic infection process and rapid disease progression. This pathogen can persist in the soil for 5–10 years, making eradication difficult. During nursery cultivation, asymptomatic Eucalyptus cuttings may harbor large quantities of *R. solanacearum*, resulting in latent infections and facilitating long-distance dissemination (Swanson et al., 2007). Furthermore, *R. solanacearum* can survive in sand beds and subsequently infect mother plants, from which it may be transmitted through vegetative propagation (Mafia et al., 2012). With the expansion of Eucalyptus varieties and plantation area, bacterial wilt has become a major constraint to sustainable Eucalyptus cultivation, leading to severe economic losses in the industry (Fegan and Prior, 2005). *R. pseudosolanacearum* can infect more than 450 species of plants in more than 50 families, including Eucalyptus, Casuarina, mulberry, tomatoes, potatoes, peanuts and so on. It is spread mainly through contaminated plants, soil, water, and farm tools (He et al., 2020; Lopes and Rossato, 2018; Okiro et al., 2019). At present, no effective measures have been established worldwide for eradicating this disease (Ran et al., 2005). Therefore, rapid and sensitive detection of *R. pseudosolanacearum* is pivotal to effective control and diagnosis of the disease.

Currently, the identification of *Ralstonia pseudosolanacearum* relies on isolation and culture, immunological assays, and molecular diagnostic techniques. However, conventional detection methods have certain limitations, being relatively time-consuming and labor intensive. And PCR usually requires temperature-variable amplification and gel electrophoresis. In recent years, several isothermal amplification techniques, including recombinase polymerase amplification (RPA), recombinase-aided amplification (RAA), loop-mediated isothermal amplification (LAMP), and enzymatic recombinase amplification (ERA) have been developed and widely applied in the detection various pathogenic fungi, bacteria, and viruses (Gui et al., 2025; Shao et al., 2023; Skenndri et al., 2025; Song et al., 2025; Wang et al., 2023). Compared with traditional methods, isothermal amplification techniques operating at temperatures between 37 and 60°C offer simplicity and convenience for operation, demonstrating great potential for onsite field sample detection (Chen et al., 2022; Jiang et al., 2025a). ERA represents an improved version of RPA technology. It can amplify a few target DNA fragments billions of times within minutes across a wide temperature range of 37–42°C (Xu et al., 2022). It does not require lid opening during the process, offering advantages including short reaction time, simple operation, good specificity, and high sensitivity. ERA has been successfully applied in the detection of *Mycobacterium tuberculosis* (Gan et al., 2024), feline calicivirus (Liu et al., 2022), porcine circovirus type 3 (Zhang et al., 2021) and *Chlamydia psittaci* (Jiang et al., 2025b).

Clustered regularly interspaced short palindromic repeats (CRISPRs) are DNA repeat sequences identified in prokaryotes (Mojica et al., 2005; Cong et al., 2013). CRISPR and associated Cas proteins constitute a crucial component of the adaptive immune system in archaea and bacteria, and function to defend against invasive nucleic acids from foreign plasmids and bacteriophages (Barrangou et al., 2007; Marraffini and Sontheimer, 2008). The CRISPR/Cas12a system has been extensively applied in the detection of bacteria, viruses, small-molecule peptides, and proteins (Wang B. et al., 2025). During this process, Cas12a interacts with crRNA to form a Cas12a-crRNA complex. By recognizing a short protospacer adjacent motif (PAM) within the target gene, it triggers trans-cleavage activity, thereby cutting nonspecific single-stranded DNA (ssDNA) and generating a fluorescence signal to determine the detection result (Chen et al., 2018).

The integration of CRISPR/Cas systems with isothermal amplification technology creates a highly sensitive, equipment-free detection method with strong potential for the early diagnosis and management of pathogenic bacterial infections (Fan et al., 2023; Gan et al., 2024; Liu et al., 2024). Furthermore, integrating ERA with CRISPR/Cas12a has been proven to increase detection efficiency, offering a promising solution for onsite testing in resource-limited settings (Ding et al., 2023).

In this study, we developed a novel ERA-CRISPR/Cas12a detection system targeting the highly conserved *egl* gene of *R. pseudosolanacearum*. The method involved rational design of ERA primers and crRNA specific to this conserved genomic region, establishing a sensitive and specific molecular diagnostic platform. The detection results can be determined by fluorescence intensity, color under blue light and LFS color. This innovative approach enables rapid, sensitive, specific and visual detection *R. pseudosolanacearum* in Eucalyptus samples.

## Materials and methods

### Reagents

The basic nucleic acid amplification kit (ERA), LbCas12a, the F-B reporter probe, and LFS were obtained from GenDx Biotech Co., Ltd. (Suzhou, China). A Bacteria DNA Isolation Mini Kit was purchased from Vazyme Biotechnology (Nanjing, China). The fluorescent probe (F-Q) and 2×Taq PCR mix were purchased from Sangon Biotech Co., Ltd. (Shanghai, China). The pBM23 vector, prep midi plasmid kit and DH5α cells were obtained from Xinkailai Biotechnology Co., Ltd. (Guangzhou, China).

### Strains and DNA extraction

A total of 20 strains, including 3 *R. pseudosolanacearum* strains and 17 nontargeting pathogenic bacteria, were used in this study. The strains are detailed in **Table 1**. The nontargeting bacteria included *Bacillus subtilis*, *Burkholderia cepacia*, *Klebsiella pneumoniae*, *Paenibacillus favisporus*, *Pectobacterium actinidiae*, *Pseudomonas jinjuensis*, *Priestia megaterium*, *Pseudomonas putida*, *Ralstonia mannitolilytica*, *Serratia marcescens*, *Xanthomonas axonopodis*, and *Xanthomonas citri*subsp*. citri*. These materials were provided by the Forest Protection Laboratory of South China Agricultural University and our laboratory. The *R. pseudosolanacearum* isolates were obtained from Yingde city, Guangdong Province. All the isolates were cultured at 28°C under dark conditions for 24–48 hours and identified through 16S rRNA gene sequencing. Bacterial DNA was extracted using the Bacteria DNA Isolation Mini Kit and stored at -20°C for future use.

**Table 1:** Information on the strains used in this study.

Number	Species	Origin	ERA-CRISPR/Cas12a^a^
1	*Ralstonia pseudosolanacearum* Rs	Eucalyptus	+
2	*Ralstonia pseudosolanacearum* Rs1	Eucalyptus	+
3	*Ralstonia pseudosolanacearum* Rs2	Eucalyptus	+
4	*Bacillus subtilis* Bs	Soil	–
5	*Bacillus subtilis* Bs1	Soil	–
6	*Bacillus subtilis* Bs2	Soil	–
7	*Burkholderia cepacia* Bc	Eucalyptus	–
8	*Klebsiella pneumoniae* Kp	Eucalyptus	–
9	*Paenibacillus favisporus* Pf	Soil	–
10	*Paenibacillus favisporus* Pf1	Soil	–
11	*Paenibacillus favisporus* Pf2	Soil	–
12	*Pectobacterium actinidiae* Pa	Soil	–
13	*Pseudomonas jinjuensis* Pj	Soil	–
14	*Priestia megaterium* Pm	Soil	–
15	*Pseudomonas putida* Pp	Eucalyptus	–
16	*Ralstonia mannitolilytica* Rm	Water	–
17	*Serratia marcescens* Sm	Eucalyptus	–
18	*Xanthomonas axonopodis* Xa	Eucalyptus	–
19	*Xanthomonas axonopodis* Xa1	Eucalyptus	–
20	*Xanthomonas citri*subsp*.citri* Xc	Soil	–

a Positive (+) or negative (-) reaction results in the ERA-CRISPR/Cas12a assay for detecting *R. pseudosolanacearum*.

### Primer and crRNA design and ssDNA reporter

**Figure 1:**
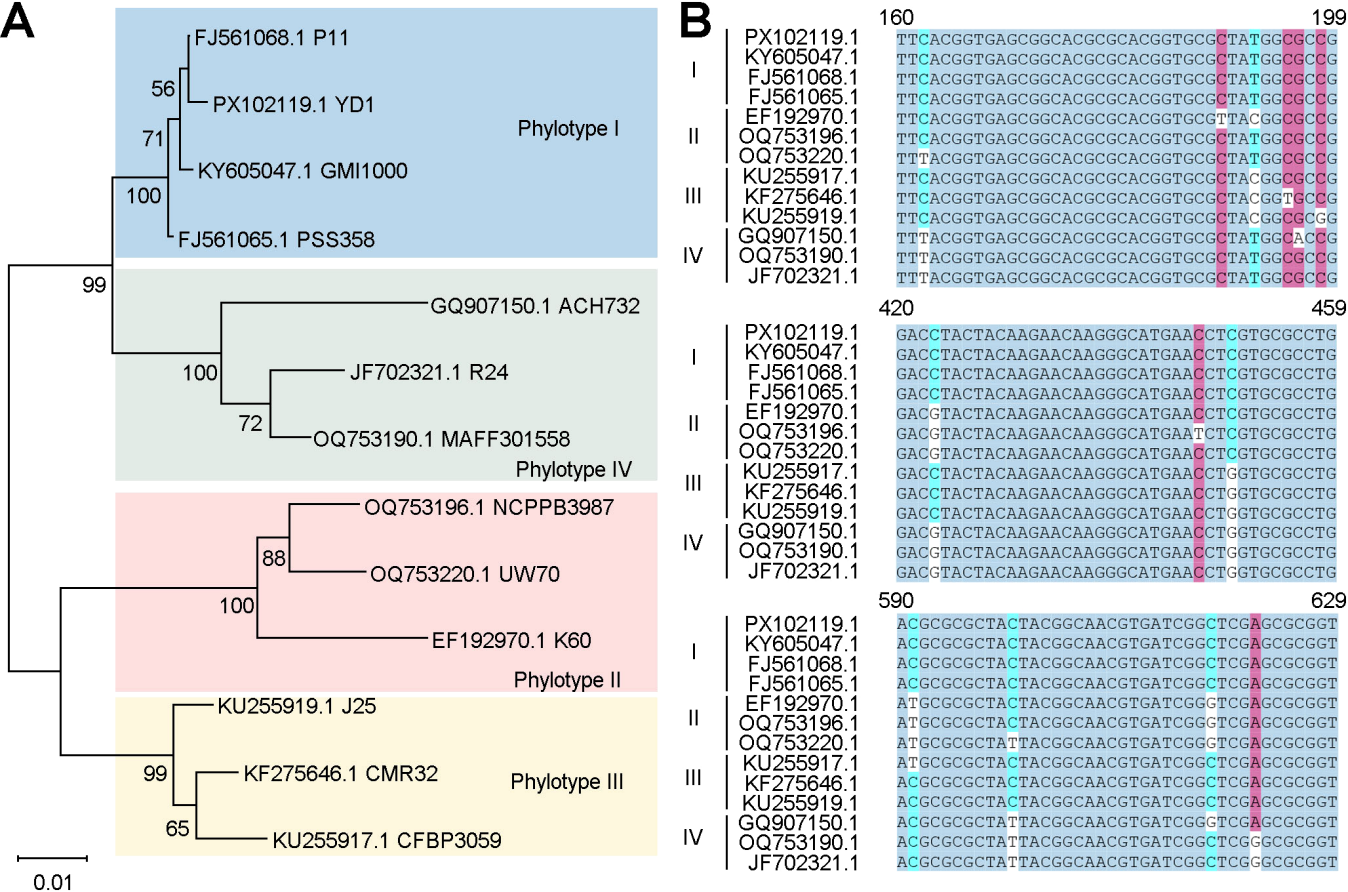
Selection of the *egl* gene. **(A)** Phylogenetic tree of the *egl* gene, constructed using 13 *egl* gene sequences from GenBank. **(B)** Sequence comparison of *egl* genes of different phylogenetic types of the *R. solanacearum sensu lato* species complex.

**Figure 2:**
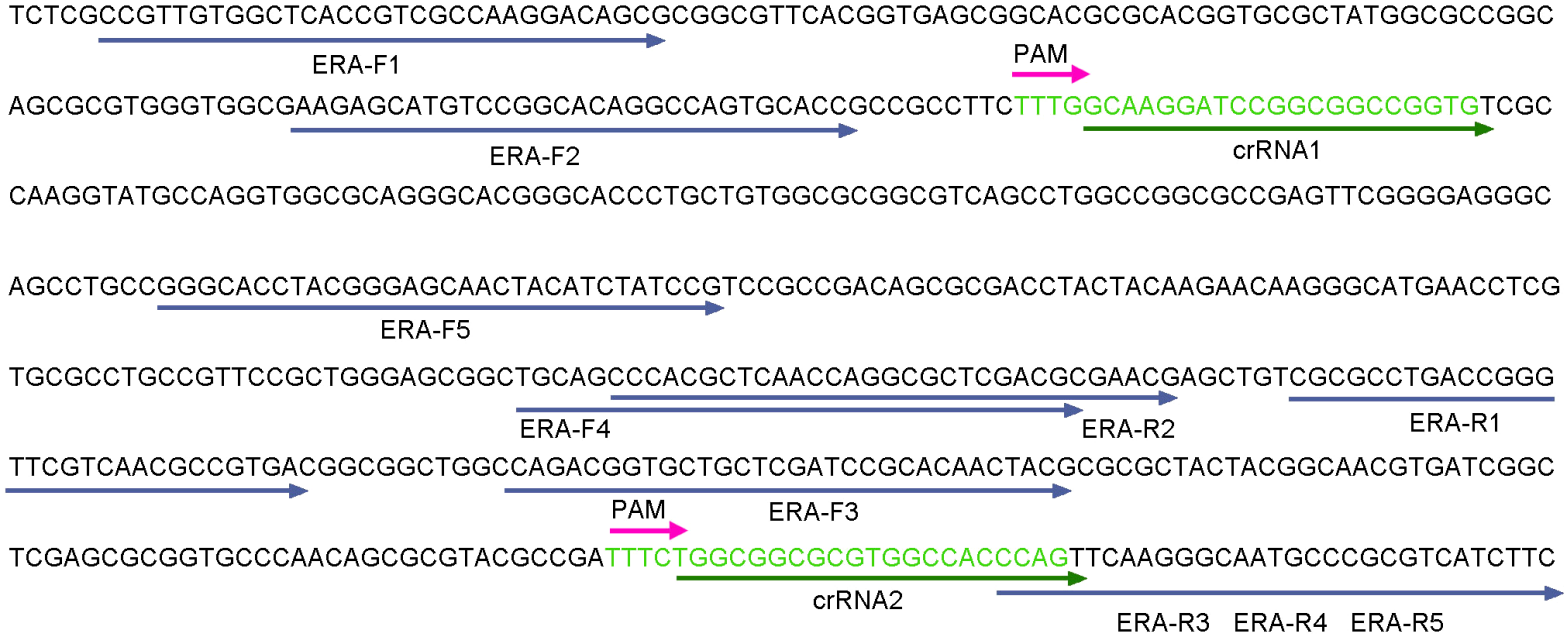
Design of the ERA primers, crRNA and PAM in the *egl* gene.

The *egl* gene of *R. pseudosolanacearum* is highly conserved (**Figure 1**). In this study, the *egl* (PX102119.1) gene was selected as the target for designing ERA primers (ERA-F1/R1, ERA-F2/R2, ERA-F3/R3, ERA-F4/R4, and ERA-F5/R5) using Primer 5.0 software (**Table 2**). Additionally, crRNA sequences were designed utilizing the CHOPCHOP online tool (http://chopchop.cbu.uib.no/) (**Figure 2**). The principle of crRNA designation is as follows: crRNA sequencing must target the conserved region of the ERA amplification product (**Table 2**). All the primers used in the present study were synthesized by Sangon Biotech (Shanghai, China). A fluorescent probe (F-Q) was selected, which was labeled with FAM at the 5’ end and a BHQ1 quenching group at the 3’ end. For the lateral flow reporter probe, the 5’ end was labeled with FAM fluorescent dye, and the 3’ end was labeled with biotin (**Table 2**).

**Table 2:** The primers and probes used in ERA-CRISPR/Cas12a system.

Name	Sequence (5’ to 3’)	Method
crRNA1	UAAUUUCUACUAAGUGUAGAU GCAAGGATCCGGCGGCCGGTG	ERA-CRISPR/Cas12a assay
crRNA2	UAAUUUCUACUAAGUGUAGAU TGGCGGCGCGTGGCCACCCAG	
F1	ACCGACACCACGACCCTGAA	PCR amplification
R1	GTTCATCAGCCCGAAGATGA	
ERA-F1	CCGTTGTGGCTCACCGTCGCCAAGGACAGC	For ERA
ERA-R1	TCACGGCGTTGACGAACCCGGTCAGGCGCG	
ERA-F2	AAGAGCATGTCCGGCACAGGCCAGTGCACC	
ERA-R2	GTTCGCGTCGAGCGCCTGGTTGAGCGTGGG	
ERA-F3	CAGACGGTGCTGCTCGATCCGCACAACTAC	
ERA-R3	AAGATGACGCGGGCATTGCCCTTGAACTGG	
ERA-F4	TGCAGCCCACGCTCAACCAGGCGCTCGACG	
ERA-R4	AAGATGACGCGGGCATTGCCCTTGAACTGG	
ERA-F5	GGGCACCTACGGGAGCAACTACATCTATCC	
ERA-R5	AAGATGACGCGGGCATTGCCCTTGAACTGG	
ssDNA-FQ	FAM-TTATT-BHQ1	Fluorescence-based probe
ssDNA-FB	FAM-TTATT-Biotin	Paper-based lateral flow strips probe

### Screening of the ERA primers and optimizing the ERA detection system

The total volume of the ERA reaction mixture was 50 μL, consisting of 20 μL of deliquescent agent, 2.5 μL each of 10 μM forward and reverse primers for ERA, 1 μL of DNA template, 2 μL of activator and 22 μL of RNase-free H_2_O. The mixture was incubated in a water bath at 38°C for 20 minutes. In accordance with the instructions of the basic nucleic acid amplification kit (ERA), five pairs of primers (ERA-F1/R1, ERA-F2/R2, ERA-F3/R3, ERA-F4/R4 and ERA-F5/R5) were combined with crRNA1 and crRNA2 to construct the ERA-CRISPR/Cas12a reaction system, in which ERA-F1/R1, ERA-F2/R2 and crRNA1 are combined, and ERA-F3/R3, ERA-F4/R4, ERA-F5/R5 and crRNA2 were combined respectively. The optimal combination was selected on the basis of the fluorescence amplification curve and final fluorescence intensity. RNase-free H_2_O served as the negative control. All experiments were performed in triplicate.

To improve the amplification efficiency of the ERA amplification system, we systematically optimized the temperature and duration of the ERA reaction. The primer combination ERA-F3/R3 and crRNA2, identified as optimal in previous experiments, was used as a template. The reaction was conducted at a series of temperatures—35, 36, 37, 38, 39, 40, 41, 42, 43, 44 and 45°C, and then the amplification times were set to 10, 15, 20, 25 and 30 min, respectively. RNase-free H_2_O was used as the negative control. By comprehensively analyzing the trends in the fluorescence curves, fluorescence intensity and LFS color results, the optimal ERA reaction temperature and time were determined.

### Establishment and optimization of ERA-CRISPR/Cas12a

The reaction mixture of the ERA-CRISPR/Cas12a system contained 0.4 μL of 10 μM LbCas12a, 0.2 μL of 20 μM crRNA, 2 μL of 10× buffer, 0.15 μL of 100 μM fluorescent probe, and 5 μL of the ERA amplification product, with RNase-free H_2_O added to establish a final volume of 20 μL. For the LFS method, its composition was the same as that of the fluorescence reaction system, except that the fluorescence probe was replaced by biotin probe.

To improve the efficiency of trans-cleavage of LbCas12a, the concentration ratio of LbCas12a to crRNA was adjusted. The LbCas12a/crRNA ratios were set at 1:1, 1:2, 1:3, 1:4 and 1:5, with the concentration of LbCas12a being 100 nM and that of crRNA being 100, 200, 300, 400, and 500 nM, respectively. Afterward, the concentration of crRNA was maintained at 100 nM, with LbCas12a concentrations of 200, 300, 400, and 500 nM. The ratios of LbCas12a to crRNA were 2:1, 3:1, 4:1, and 5:1, and the best combination was selected according to the fluorescence intensity. Furthermore, we optimized the concentration of the ssDNA probe to 200, 400, 600, 800 and 1000 nM. The other conditions remained unchanged, and RNase-free H_2_O was used as the negative control. This test was repeated three times.

### Specificity and sensitivity of the ERA/CRISPR-Cas12a detection system

A total of 20 strains including 3 *R. pseudosolanacearum* strains and 17 nontargeting bacteria, were used to assess the specificity of the ERA-CRISPR/Cas12a system. With RNase-free H_2_O as a negative control, this experimental process was repeated three times.

To evaluate the limit of detection of the ERA-CRISPR/Cas12a detection system, we constructed a recombinant plasmid according to the highly conserved gene *egl* of *R. pseudosolanacearum*. First, a pair of primers, F1 and R1 (**Table 2**), was designed for PCR amplification, and the resulting PCR products were subsequently purified. The purified target fragment was cloned and inserted into the pBM23 vector and transformed into DH5α cells for overnight culture. Finally, the recombinant plasmid was extracted using a Prep Midi Plasmid Kit and verified through DNA sequencing. The recombinant plasmid was measured using a spectrophotometer (Thermo Fisher Scientific, Madison, USA) and diluted 10-fold in RNase-free H_2_O to obtain a series of concentrations ranging from 10^6^ to 10–^2^ copies/μL. The copy number was calculated as follows: copies/μL = 6.02×10^23^×concentration (ng/μL) 10^-9^/(number of plasmid bases × 660). All the plasmid solutions were stored at -20°C until use. The detection limit of the ERA-CRISPR/Cas12a fluorescence and LFS system was determined on the basis of the observation results. All experiments were performed with at least three replicates, with RNase-free H_2_O used as a negative control.

### Detection of *R. pseudosolanacearum* in field samples

To verify the ability of the proposed method for real sample applications, we collected 26 Eucalyptus samples from the field for analysis (**Table 3**). Genomic DNA was extracted by the Bacteria DNA Isolation Mini Kit. The DNA samples were tested using the final ERA-CRISPR/Cas12a-fluorescence and ERA-CRISPR/Cas12a LFS. Moreover, the same samples were analyzed by PCR. The genomic DNA of *R. pseudosolanacearum* and nuclease-free H_2_O were used as positive and negative controls, respectively. The reaction mixture (25 μL) for PCR contained 12.5 μL of 2×Taq PCR mix, 0.5 μL each of F1/R1 (10 μM) (**Table 2**), 1 μL of DNA template and 10.5 μL of nuclease-free H_2_O. The reaction conditions were as follows: predenaturation at 94°C for 5 min; 30 cycles of denaturation at 95°C for 1 min, annealing at 56.7°C for 50 s, and extension at 72°C for 1 min; and a final extension at 72°C for 5 min.

**Table 3:** The detection results of 26 field samples.

Samples	Origin	Detection results^a^	
		ERA-CRISPR/Cas12a	PCR
1	Eucalyptus tissue	–	–
2	Eucalyptus tissue	+	–
3	Eucalyptus tissue	+	+
4	Eucalyptus tissue	+	+
5	Eucalyptus tissue	–	–
6	Eucalyptus tissue	–	–
7	Eucalyptus tissue	+	+
8	Eucalyptus tissue	+	+
9	Eucalyptus tissue	+	+
10	Eucalyptus tissue	+	+
11	Eucalyptus tissue	+	+
12	Eucalyptus tissue	+	+
13	Eucalyptus tissue	+	+
14	Eucalyptus tissue	+	+
15	Eucalyptus tissue	+	+
16	Eucalyptus tissue	+	+
17	Eucalyptus tissue	+	+
18	Eucalyptus tissue	+	+
19	Eucalyptus tissue	+	+
20	Eucalyptus tissue	+	+
21	Eucalyptus tissue	–	–
22	Eucalyptus tissue	+	+
23	Eucalyptus tissue	+	+
24	Eucalyptus tissue	+	+
25	Eucalyptus tissue	+	+
26	Eucalyptus tissue	+	+

a Positive (+) or negative (-) reaction results for detecting *R. pseudosolanacearum*.

### Readouts

The detection results were obtained by fluorescence intensity, LFS and color under blue light. The amplification curve and the final fluorescence intensity were read using real-time quantitative PCR, and the fluorescence signal was collected every minute at 38 °C for 30 min. For the ERA-LFS assay, the FAM-Biotin-labeled amplification products will be captured by streptavidin-coated gold nanoparticles. These gold nanoparticles accumulate at the T line, generating red bands that are clearly visible with the naked eye. The amplification product was diluted with RNase-free H_2_O, and the dipsticks were inserted into the mixture within 2 minutes at room temperature. The presence of T and C red lines indicated a positive result, whereas the absence of a red T line indicated a negative result. If the C line was not present, the result was not credible. Moreover, we used a blue illuminator to observe the results, in which the presence of green fluorescence served as an indicator of the presence of target DNA.

### Statistical analysis

Statistical analyses and graphic design were performed using GraphPad Prism, and the results are presented as the mean ± standard deviation (SD). One-way ANOVA was employed to examine differences among multiple groups. All comparisons were considered statistically significant at P < 0.05 and highly significant at P < 0.01.

## Results

### The workflow and feasibility of the ERA-CRISPR/Cas12a platform

**Figure 3:**
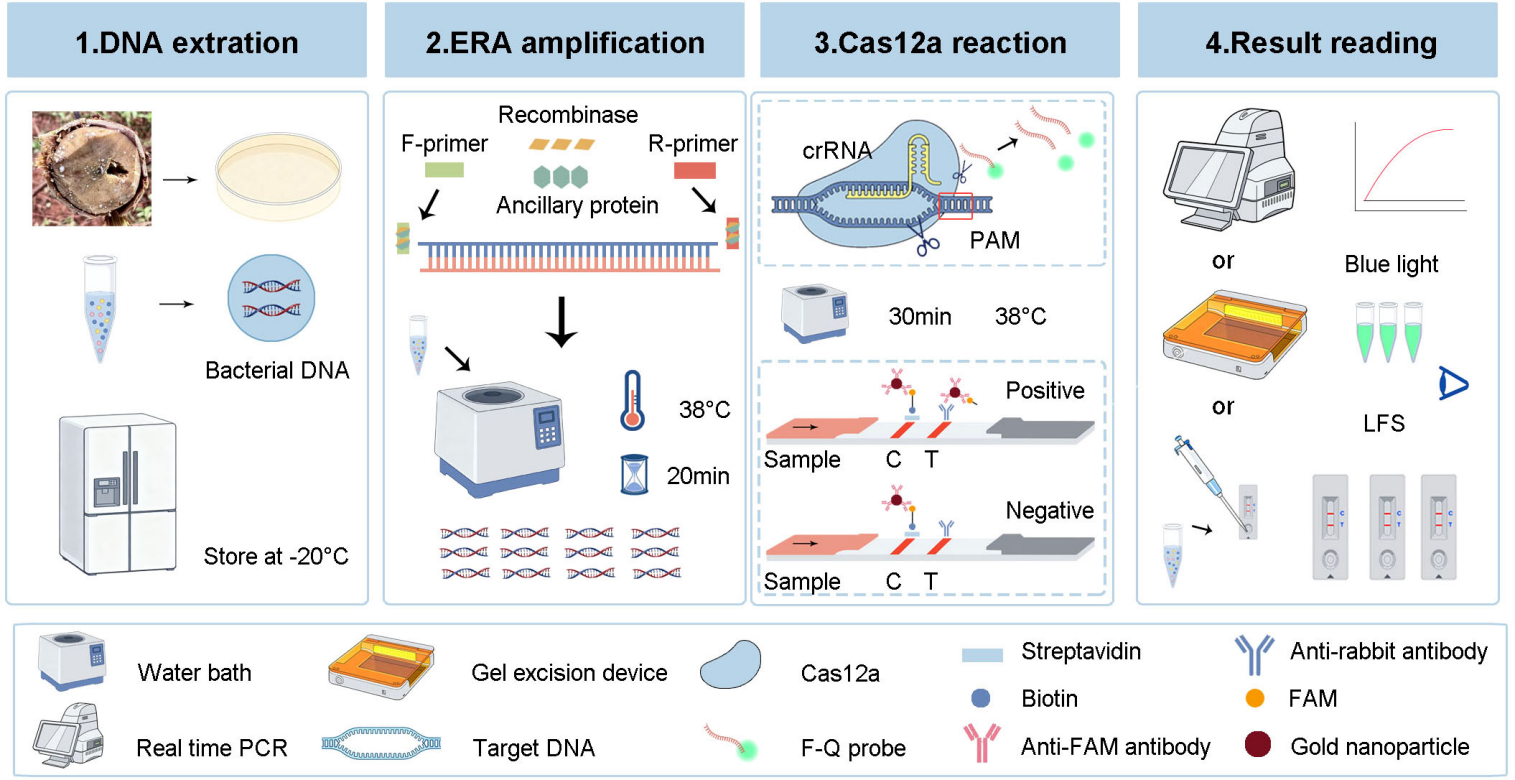
Workflow of the ERA-CRISPR/Cas12a detection platform. Firstly, the DNA of the bacteria was extracted and stored at -20°C. Secondly, the ERA amplification was performed at 38°C for 20 min to generate a large number of amplification products. Thirdly, the ERA products were added to CRISPR/Cas12a fluorescence and LFS system and reacted at 38°C for 30 min. Finally, the detection results were read by fluorescence intensity, LFS and color under blue light.

The detailed workflow of the ERA-CRISPR/Cas12a detection platform is shown in **Figure 3**. First, fresh samples were collected from the field, followed by purification and cultivation, after which the DNA of the bacteria was extracted. Second, the entire ERA amplification reaction was performed by incubation at 38°C for 20 min. Finally, the ERA amplification products were added to the EP tube containing the CRISPR/Cas12a fluorescence and LFS reaction system. After 30 min of reaction at 38°C, the fluorescence intensity, LFS and color under blue light were read.

**Figure 4:**
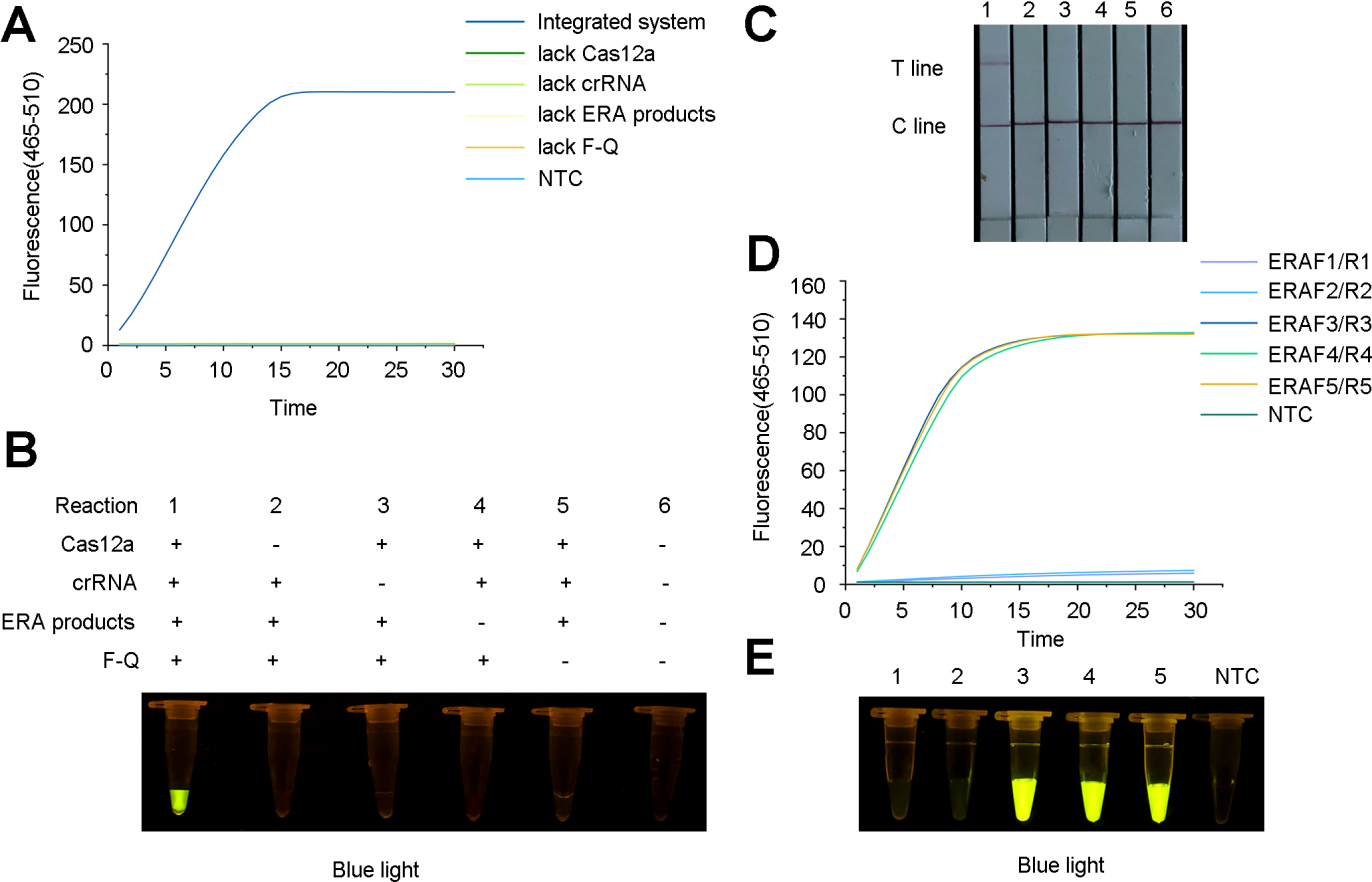
Feasibility analysis for the ERA-CRISPR/Cas12a detection system and screening for the optimal primer combinations for *Ralstonia pseudosolanacearum*. **(A)** Fluorescence curve and intensity of reaction component changed with time within 30 minutes on a qPCR instrument. “NTC”, negative control. **(B)** Fluorescence under blue light containing Cas12a, crRNA, ERA amplification products and F-Q probe demonstrated that the ERA-CRISPR/Cas12a system detected *R. pseudosolanacearum*. **(C)** Feasibility analysis of lateral flow strips (LFS) detection. 1, all components; 2-5, lacking Cas12a, crRNA, ERA amplification products and F-Q probe respectively; 6, negative control. **(D)** Fluorescence detection of primers was screened using a qPCR instrument. **(E)** Detection of green fluorescence under blue light. 1-2, ERAF1/R1, ERAF2/R2 and crRNA1; 3-5, ERAF3/R3, ERAF4/R4, ERAF5/R5 and crRNA2, respectively; NTC, negative control.

In the presence of the ERA amplification products, Cas12a, crRNA and F-Q, the fluorescence intensity significantly increased within 30 min, indicating that the constructed system could accurately identify *R. pseudosolanacearum* (**Figures 4A–C**). In the preliminary experiments, the use of the ERA primers was feasible, and Cas12a specifically recognized target genes under the guidance of crRNA, resulting in cleavage activity.

### Selection of the ERA primers and optimization of detection system

To optimize the reaction system, five ERA primer sets (ERA-F1/R1, ERA-F2/R2, ERA-F3/R3, ERA-F4/R4, and ERA-F5/R5) and two crRNA primer sets were designed and combined. The results revealed that the use of ERA-F3/R3 in conjunction with crRNA2 resulted in a faster fluorescence amplification rate and higher fluorescence intensity (**Figure 4D**). The fluorescence under blue light also showed the same result (**Figure 4E**). Therefore, the ERA-F3/R3 primer and crRNA2 were selected as the best primer pairs.

**Figure 5:**
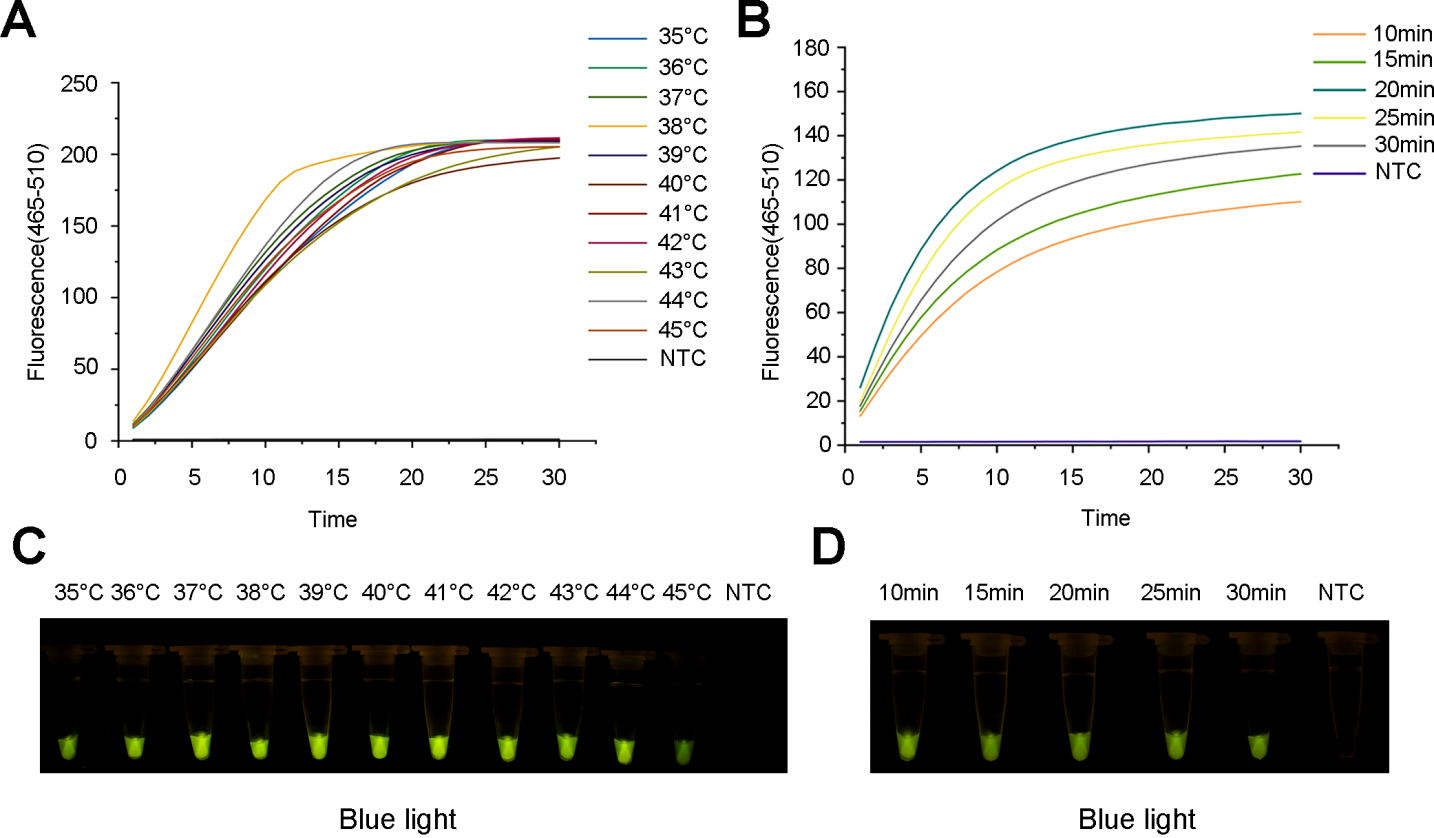
Optimization of the ERA reaction for the ERA-CRISPR/Cas12a assay. **(A)** Fluorescence curve and intensity of ERA reaction temperatures. **(B)** Fluorescence curve and intensity of ERA reaction times. **(C)** Detection of visible green fluorescence for ERA reaction temperatures under blue light. **(D)** Detection of visible green fluorescence for ERA reaction times under blue light. NTC, negative control.

Furthermore, the optimal reaction temperature and time of the ERA system were determined. The results indicated that the fluorescence amplification rate was maximized at 38 °C. Additionally, the most rapid and highest fluorescence signals were observed after 20 min of incubation. Therefore, clear and high fluorescence intensity was observed in the reaction with 20 min of incubation at 38°C (**Figures 5A, B**), which was consistent with the observation under blue light (**Figures 5C, D**).

### Optimization of the ERA-CRRSPR/Cas12a detection system

**Figure 6:**
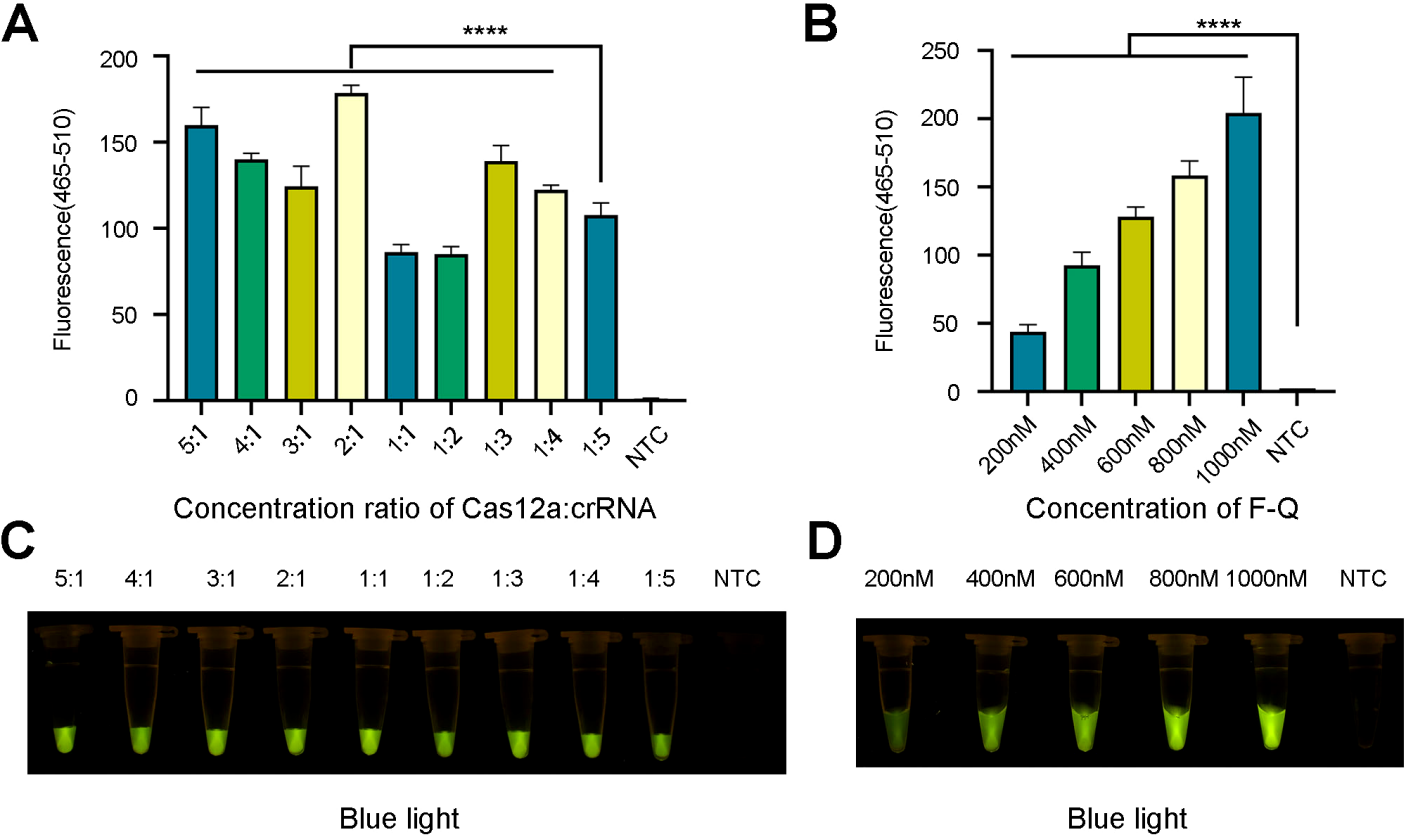
Optimization of fluorescence system. **(A, C)** The ratio optimization of Cas12a and crRNA. **(B, D)** The concentration optimization of ssDNA reporter. The results came from three independent experiments and the bars were represented as the means ± SD. P<0.0001 (^****^). NTC, negative control. **(A, B)** Fluorescence detection used qPCR machine. **(C, D)** Visible fluorescence detection under blue light.

After the best combination of pairs was selected, the optimal concentration ratios of Cas12a to crRNA (5:1, 4:1, 3:1, 2:1, 1:1, 1:2, 1:3, 1:4, and 1:5) and concentrations of F-Q probes (200, 400, 600, 800, and 1000 nM) were investigated. When the concentration ratio of Cas12a to crRNA was 2:1 (that is, when the two concentrations were 100 nM and 200 nM), the fluorescence curve and intensity were the highest (**Figures 6A, C**). Similarly, when the concentration of the F-Q probe reached 1000 nM, the fluorescence intensity was the highest (**Figures 6B, D**). Considering the cost of the reagents, 800 nM of ssDNA was chosen for the subsequent experiments.

**Figure 7:**
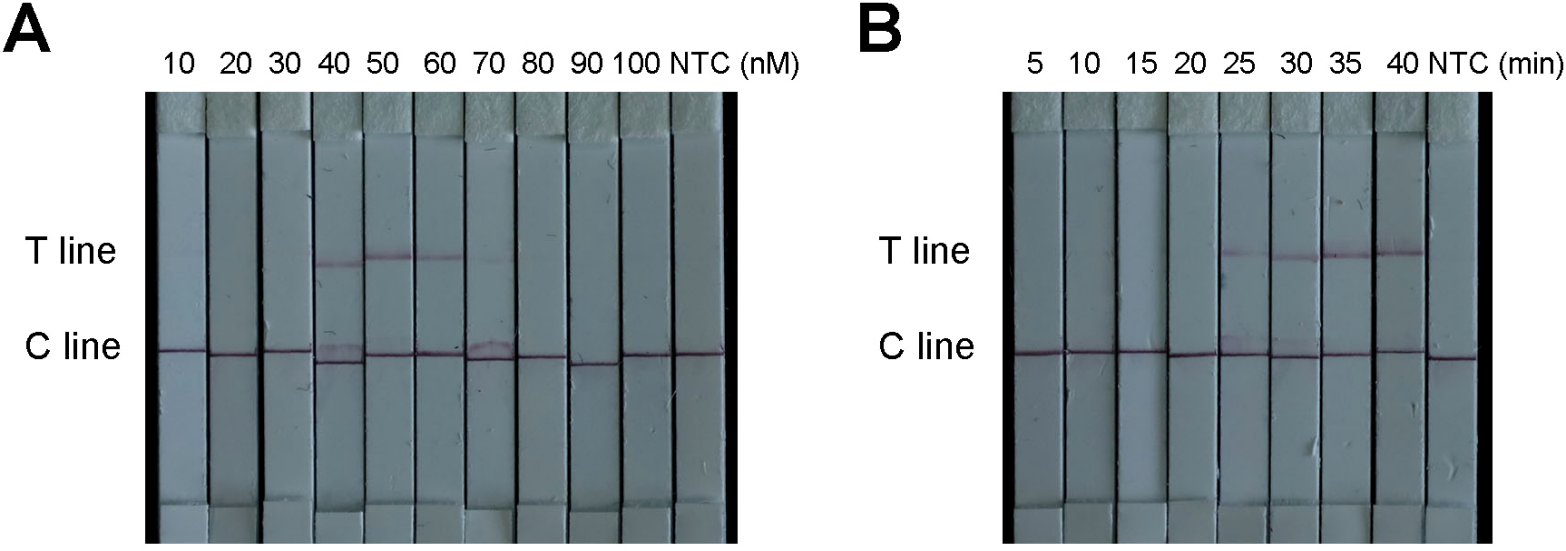
Optimization of the ERA-CRISPR/Cas12a based diagnostic lateral-flow detection. **(A)** Optimization of the F-B probe concentration. **(B)** Optimization of LFS reaction time. “NTC” was negative control.

With respect to the lateral flow test strip detection method, both excessively low and high concentrations of the FAM-TTATT-Biotin ssDNA reporter affected the results. However, when the biotin probe concentration was 10, 20, 30, 70, 80, 90, or 100 nM, the T line was weak or even absent. Therefore, 50 nM was selected as the optimal concentration for the biotin probe (**Figure 7A**). Furthermore, the incubation time of the ERA-CRISPR/Cas12a system was also optimized. The T line was already clearly visible after a reaction time of 30 min. Therefore, an incubation period of 30 min is recommended as optimal for this test strip system (**Figure 7B**).

### Specificity and sensitivity analysis of the established ERA-CRISPR/Cas12a system

**Figure 8:**
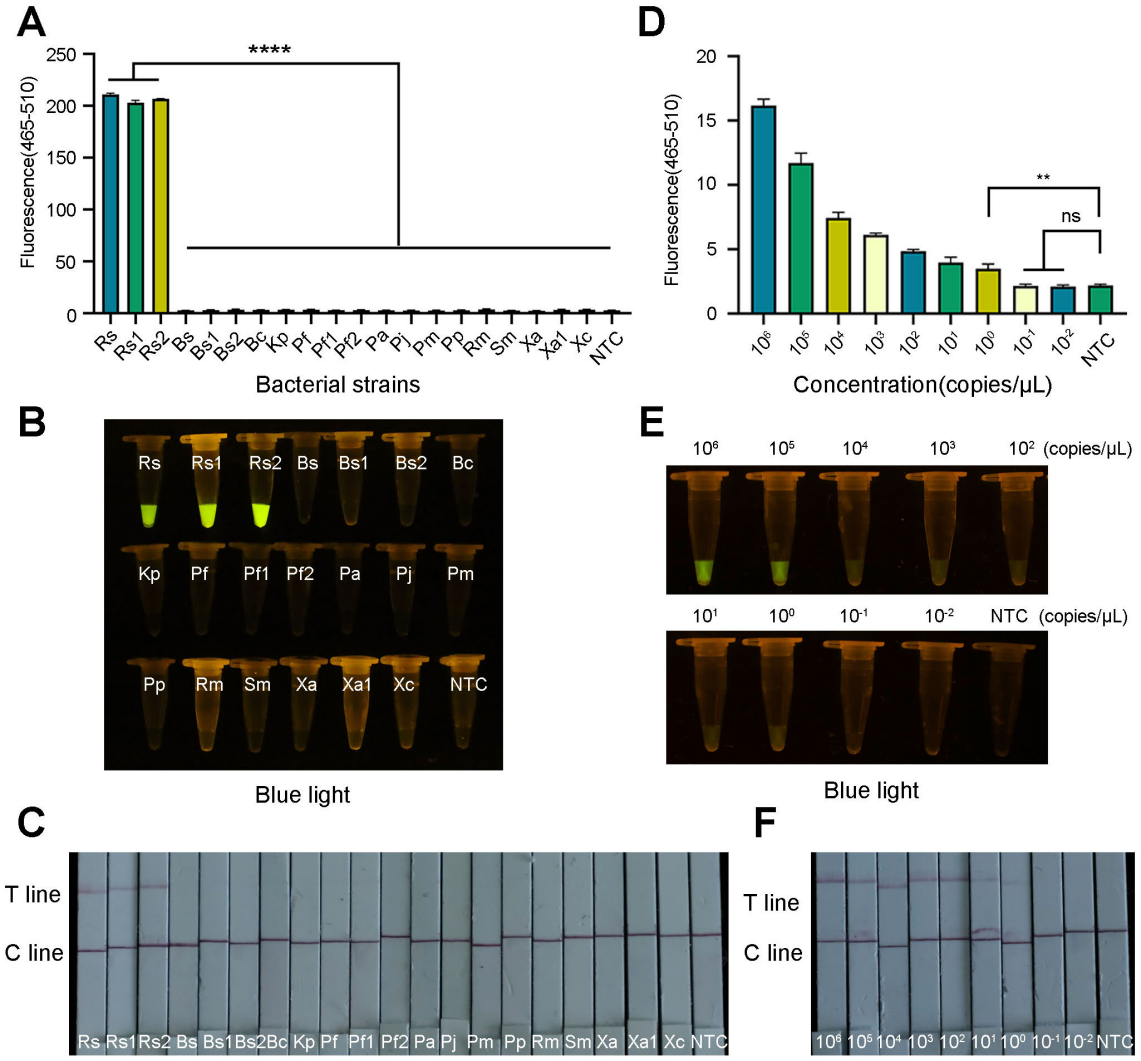
Specificity and sensitivity of the ERA-CRISPR/Cas12a system. **(A)** Specificity tests were performed to determine the fluorescence intensity using a qPCR instrument. **(B)** Visible fluorescence detection under blue light. **(C)** Lateral-flow assay strip was used to determine the detection specificity for *R. pseudosolanacearum*. **(D)** Fluorescence curve and final fluorescence intensity of ERA-CRISPR/Cas12a detected by gradient dilution of the recombinant plasmid. **(E)** Visible fluorescence under blue light. **(F)** Sensitivity test of ERA-CRISPR/Cas12a LFS. Line T and line C indicated the positive line and control line respectively. Rs, *R. pseudosolanacearum*; Rs1, *R. pseudosolanacearum*; Rs2, *R. pseudosolanacearum*; Bs, *Bacillus subtilis*; Bs1, *Bacillus subtilis*; Bs2, *Bacillus subtilis*; Bc, *Burkholderia cepacia*; Kp, *Klebsiella pneumoniae*; Pf, *Paenibacillus favisporus*; Pf1, *Paenibacillus favisporus*; Pf2, *Paenibacillus favisporus*; Pa, *Pectobacterium actinidiae*; Pj, *Pseudomonas jinjuensis*; Pm, *Priestia megaterium*; Pp, *Pseudomonas putida*; Rm, *Ralstonia mannitolilytica*; Sm, *Serratia marcescens*; Xa, *Xanthomonas axonopodis*; Xa1, *Xanthomonas axonopodis*; Xc, *Xanthomonas citri*subsp*.citri*. The bars were represented as the means ± SD. ^****^P<0.0001. ^**^P<0.01. ns, insignificance. NTC, negative control.

To verify the specificity of the ERA-CRISPR/Cas12a system, *R. pseudosolanacearum* and 17 nontarget bacteria were tested. The results demonstrated that only the *R. pseudosolanacearum* strains produced fluorescence signals, whereas the other pathogenic bacteria and the negative control did not produce fluorescence signals (**Figure 8A**). In the LFS, only the test line was visible for *R. pseudosolanacearum*, while the C line appeared in the other samples (**Figure 8C**). In addition, only *R. pseudosolanacearum* produced significant fluorescence under blue light (**Figure 8B**). These results suggested that the ERA-CRISPR/Cas12a system is highly specific for *R. pseudosolanacearum* and does not cross-react with other pathogens. The specificity tests were repeated three times, and each repetition provided consistent results.

To evaluate the detection sensitivity of the ERA-CRISPR/Cas12a system, the constructed recombinant plasmids were serially diluted to final concentrations ranging from 10^6^ to 10–^2^ copies/μL. The fluorescence signal gradually decreased as the plasmid concentration decreased. The template at a low concentration (specifically, 10^0^ copies/μL) exhibited obvious fluorescence amplification and clear test line. A statistically significant difference was observed in comparison to the negative control (**Figure 8D**). LFS and blue light resulted in the same observations (**Figures 8E, F**). These results demonstrated that the limit of this technology could reach 10^0^ copies/μL. These results were consistent across the three replicates.

### Application of ERA-CRISPR/Cas12a in field samples

**Figure 9:**
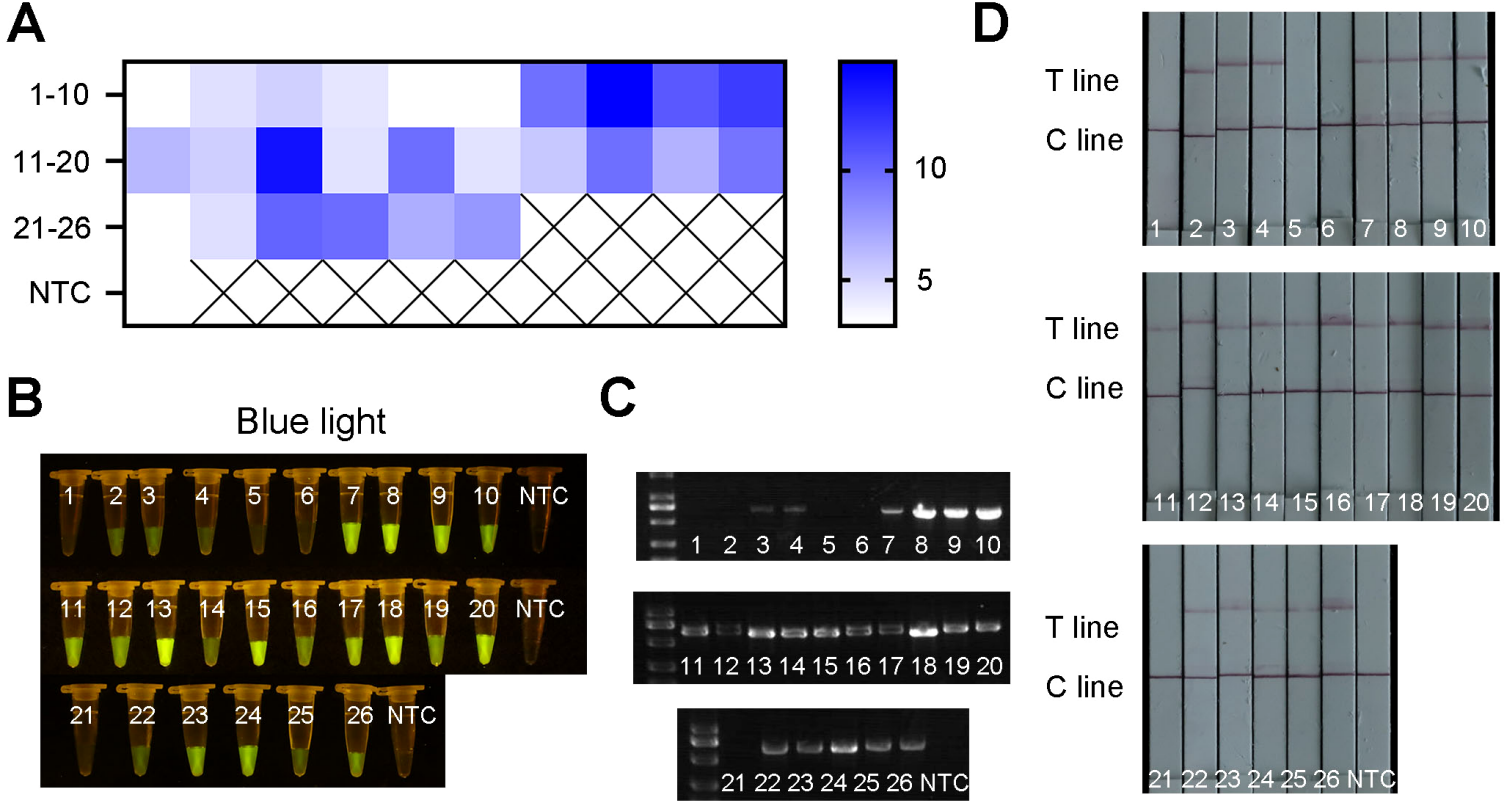
The results of field samples detection by ERA-CRISPR/Cas12a and PCR. **(A)** Fluorescence values of 26 samples using qPCR machine. **(B)** Visible fluorescence detection under blue light. **(C)** The detection results obtained by using PCR method. **(D)** Lateral-flow assay strip was used to determine the application for *R. pseudosolanacearum*.

To further evaluate the detection performance of the ERA-CRISPR/Cas12a method, 26 field samples were tested. The obtained results were subsequently compared with those obtained using the conventional PCR method. Among the 26 samples, 22 samples were identified as positive by the ERA-CRISPR/Cas12a method (**Figures 9A, B, D**), and 21 positive samples were detected by the PCR method (**Figure 9C**). These findings indicated that the detection effect of ERA-CRISPR/Cas12a was good. Its performance was comparable to, or even better than, that of the PCR method. Furthermore, the novel method could report the results within 1 hour, enabling visual detection without the need for large-scale testing instruments and improving the detection efficiency.

## Discussion

*R. solanacearum* is an important pathogen that affects Eucalyptus in many countries, posing a major health threat to Eucalyptus plants (Salanoubat et al., 2002; Santiago et al., 2015; Xiao et al., 2024). Therefore, to avoid the risk of pathogen transmission through symptomatic mother plants, establishing a new rapid detection method is essential, which is conducive to timely and effective diagnosis of potential *R. solanacearum* in the early stage and prevention of rapid spread of the pathogen and bacterial wilt. In this study, we developed a highly accurate and sensitive ERA-CRISPR/Cas12a assay for the detection of *R. pseudosolanacearum.* It was reported for the first time for detection of *R. pseudosolanacearum* in Eucalyptus. And compared to the method for detecting *Rhizoctonia cerealis* (Wang Y. X. et al., 2025), we could analysis the result under blue light, offering a more straightforward approach.

ERA is a novel isothermal amplification technique that involves rapid reactions (15–30 min) (Xie et al., 2025; Zeng et al., 2025). The most prominent feature of the ERA is its ability to amplify a large number of target DNA fragments at low temperatures without the need for sophisticated instruments. Therefore, ERA is considered a promising isothermal amplification technology and has attracted a great deal of attention from researchers. Notably, the designed primers and concentrations strongly influence the specificity and sensitivity of ERA amplification. In this study, we designed ERA primers targeting the *egl* gene, which was previously shown to be highly conserved in *R. pseudosolanacearum*, for molecular identification. The best primers were screened to satisfy the amplification demand. To further improve the specificity and sensitivity, we integrated ERA into the CRISPR/Cas12a system to detect *R. pseudosolanacearum* rapidly and accurately. ERA-CRISPR/Cas12a demonstrated high specificity and did not cross-react with other 17 nontarget pathogens. These findings indicated that the ERA primers are highly specific. Validating more robust specificity with closely related strains of *R. pseudosolanacearum* remains a key objective for future research. The ERA products can be highly specifically recognized by the crRNA of CRISPR/Cas12a. The entire basic ERA operation procedure can be completed within 20 min at a constant temperature of 38°C. After the CRISPR reaction components were added, the results were observed after 30 min of reaction and found to be similar to those reported in the literature. Liu et al. (2022) reported that the optimal reaction conditions of ERA for Feline calicivirus were at 40°C for 30 min. Ding et al. (2023) reported that the entire detection process of HPV16/18 could be completed within 25 min at a constant low temperature (35–43°C). In another study, a fluorescence system and a lateral flow system were used to detect *Chlamydia pneumonia* at 30 minutes and 15 minutes, respectively, using an ERA-CRISPR/Cas12a assay (Zhou et al., 2024). These results underscore the remarkable efficiency and speed of this method.

The detection limit of ERA-CRISPR/Cas12a in our study was 10^0^ copies/μL, which was consistently confirmed in the fluorescence and LFS detection system. And it is much higher than that reported for the PCR method (Li et al., 2019). Previously, a nucleic acid amplification technology using the LAMP method for the detection of *R. solanacearum* was developed, but with limits of 10^4–^10^6^ cells/mL (Lenarčič et al., 2014) and 10^4–^10^6^ CFU/mL (Kubota et al., 2008). In several previous studies, ERA-CRISPR/Cas12a assays were reported to be sensitive to single-digit copy numbers (Liang et al., 2025; Yang et al., 2025). These findings suggest that the ERA-CRISPR/Cas12a method is highly sensitive. Thus, the ERA-CRISPR/Cas12a method offers great potential in the detection of *R. pseudosolanacearum*, especially for samples with relatively low concentrations.

In field sample detection results of the ERA-CRISPR/Cas12a method using 25 samples, we confirmed that the results were almost identical to those of conventional PCR. In addition, the novel ERA-CRISPR/Cas12a method has higher sensitivity, compared with traditional PCR. The ERA-CRISPR/Cas12a detection system could detect the target in samples with low bacterial concentrations where the PCR method failed under the same conditions. And it demonstrated higher sensitivity compared to the PCR detection method. These findings indicated the stability and reliability of ERA-CRISPR/Cas12a. Compared with conventional PCR, ERA-CRISPR/Cas12a can quickly confirm the results with the naked eye (Deng et al., 2022; Liu et al., 2021). Furthermore, results can be visualized through test strips or under blue light because of the use of a fluorescent labeled probe. Therefore, the ERA-CRISPR/Cas12a method offers the application potential for onsite detection of pathogenic bacteria without the need for large-scale instrumentation and equipment. The entire diagnostic process of the ERA-CRISPR/Cas12a method can be completed within one hour, which is much faster than the conventional PCR method, which requires at least 2 h (Zhou et al., 2024), and the traditional culture method, which requires 48 h. The method is suitable for rapid detection of pathogens in the field or in poorly equipped laboratories via a portable thermostatic device. Overall, the ERA-CRISPR/Cas12a fluorescence detection system is suited for implementation in resource-abundant laboratories. Conversely, the LFS method or the blue light irradiation approach is more appropriate for point-of-care applications and laboratories in resource-limited environments. It is reported that one-pot ERA-CRISPR assays for on-site detection in genetically modified crops, amplifying the target in a single sealed tube, represents a significant leap forward in detection method (Liang et al., 2025). A systematic comparison between our established method and one-pot ERA-CRISPR assays will be essential to rigorously benchmark the practical benefits. And it constitutes a primary objective for our subsequent research.

## Conclusion

In summary, we developed for the first time a novel detection system for *R. pseudosolanacearum* in Eucalyptus on the basis of the ERA-CRISPR/Cas12a method. Compared with traditional detection methods, the ERA-CRISPR/Cas12a method confers the advantages of speed, ease, and high sensitivity and specificity. Moreover, it is easy to use without sophisticated equipment at room temperature. It is especially suitable for rapid on-site screening of large quantities of Eucalyptus samples. In future studies, the ERA-CRISPR/Cas12a platform could be widely used for rapid detection of *R. pseudosolanacearum*, preventing the widespread dissemination of Eucalyptus bacterial wilt.

## Data Availability

The original contributions presented in the study are included in the article. The names of the repository/repositories and accession number(s) can be found below: https://www.ncbi.nlm.nih.gov/genbank, accession number PX102119.1. Further inquiries can be directed to the corresponding author.
